# Transcranial current stimulation in epilepsy: A systematic review of the fundamental and clinical aspects

**DOI:** 10.3389/fnins.2022.909421

**Published:** 2022-08-25

**Authors:** Sara Simula, Maëva Daoud, Giulio Ruffini, Maria Chiara Biagi, Christian-G. Bénar, Pascal Benquet, Fabrice Wendling, Fabrice Bartolomei

**Affiliations:** ^1^Aix Marseille Univ, INSERM, INS, Int Neurosci Syst, Marseille, France; ^2^Neuroelectrics Barcelona, Barcelona, Spain; ^3^Univ Rennes, INSERM, LTSI-U1099, Rennes, France; ^4^APHM, Timone Hospital, Epileptology and Cerebral Rhythmology, Marseille, France

**Keywords:** transcranial current stimulation, epilepsy, transcranial electric stimulation, drug-resistant epilepsy (DRE), brain electric field, brain network, neuromodulation

## Abstract

**Purpose:**

Transcranial electrical current stimulation (tES or tCS, as it is sometimes referred to) has been proposed as non-invasive therapy for pharmacoresistant epilepsy. This technique, which includes direct current (tDCS) and alternating current (tACS) stimulation involves the application of weak currents across the cortex to change cortical excitability. Although clinical trials have demonstrated the therapeutic efficacy of tES, its specific effects on epileptic brain activity are poorly understood. We sought to summarize the clinical and fundamental effects underlying the application of tES in epilepsy.

**Methods:**

A systematic review was performed in accordance with the PRISMA guidelines. A database search was performed in PUBMED, MEDLINE, Web of Science and Cochrane CENTRAL for articles corresponding to the keywords “*epilepsy AND (transcranial current stimulation OR transcranial electrical stimulation)*”.

**Results:**

A total of 56 studies were included in this review. Through these records, we show that tDCS and tACS epileptic patients are safe and clinically relevant techniques for epilepsy. Recent articles reported changes of functional connectivity in epileptic patients after tDCS. We argue that tDCS may act by affecting brain networks, rather than simply modifying local activity in the targeted area. To explain the mechanisms of tES, various cellular effects have been identified. Among them, reduced cell loss, mossy fiber sprouting, and hippocampal BDNF protein levels. Brain modeling and human studies highlight the influence of individual brain anatomy and physiology on the electric field distribution. Computational models may optimize the stimulation parameters and bring new therapeutic perspectives.

**Conclusion:**

Both tDCS and tACS are promising techniques for epilepsy patients. Although the clinical effects of tDCS have been repeatedly assessed, only one clinical trial has involved a consistent number of epileptic patients and little knowledge is present about the clinical outcome of tACS. To fill this gap, multicenter studies on tES in epileptic patients are needed involving novel methods such as personalized stimulation protocols based on computational modeling. Furthermore, there is a need for more *in vivo* studies replicating the tES parameters applied in patients. Finally, there is a lack of clinical studies investigating changes in intracranial epileptiform discharges during tES application, which could clarify the nature of tES-related local and network dynamics in epilepsy.

## Introduction

Epilepsy is one of the most common chronic neurologic disorders affecting 70 million people worldwide. It is characterized by unpredictable seizures caused by abnormal neuronal activity in the brain (Devinsky et al., 2018). One-third of epileptic patients experience seizures that are refractory to pharmacotherapy. The complications of drug-resistant epilepsy (DRE) are devastating with severely diminished quality of life. Surgical resection of the epileptogenic zone (EZ) is not always indicated, in particular for patients with multifocal or diffuse disease or those with inaccessible EZ. In addition, surgery is associated with a relatively high number of failures (Baud et al., 2018).

Consequently, alternative treatments based on neurostimulation methods represent a promising therapeutic approach decreasing cortical excitability. On the one hand, various types of invasive neuromodulation are available: Vagus Nerve Stimulation (VNS), responsive neurostimulation (RNS), and deep brain stimulation (DBS). These three therapies involve the implantation of a neurostimulator device with potential surgical complications, and uncertainty regarding the clinical benefit for the patients. VNS consists in an implanted electrode located around the left vagus nerve and an implanted generator in subclavicar region to obtain alternation of stimulations for desynchronizing network activity modulating neurotransmitter release. DBS involves electrical stimulation with pulse-train delivered chronically, intermittently in a closed loop manner using intracerebral electrodes located precisely with stereotaxic surgery. The principle of RNS is to detect ongoing epileptiform activity using a combination of electrocorticogram recording electrodes and stimulation electrodes.

On the other hand, non-invasive brain stimulation techniques have emerged since several years for epilepsy treatment with the advantage of being safe, well tolerated, and reversible. The first technique used for treating seizures refractory to pharmacotherapies was repetitive transcranial magnetic stimulation (rTMS) (Tergau et al., 1999). The technique of rTMS uses an external electromagnetic coil inducing an electrical current flow in the targeted cortical area. However, further studies reported a lack of evidence in seizure frequency decrease using this technique and a risk of inducing seizures in patients and in healthy subjects (for more information, see reviews by Tsuboyama et al., 2020; Vanhaerents et al., 2020).

The quite recent technique of transcranial current stimulation (tES) appears to be safer, less expensive, and more portable than rTMS, which also opens the potential for “at home” usage. This is a non-invasive neuromodulatory technique which modulates brain excitability by applying low intensity, controlled currents (~1 mA and <2mA) on the brain *via* scalp electrodes (Nitsche and Paulus, 2000; Modolo et al., 2018). In particular, tES protocols can be designed to either increase or decrease neural excitability. The terms anodal and cathodal stimulation are often used in the field and are a source of confusion. An anode, in the context of tES, is a scalp electrode through which conventional current (positive charge, opposite to the flow of negatively charged electrons) flows into the brain from the stimulation device. On the other hand, a cathode is an electrode through which current flows out of the brain. It has been observed that anodal stimulation over a brain area increases cortical activity (i.e., it is excitatory) while cathodal stimulation can decrease it (i.e., it is inhibitory) (Nitsche and Paulus, 2000). In epilepsy, cathodal electrodes can be placed over the epileptogenic region to inhibit the underlying tissues. When the electrical current applied is constant and unidirectional, from anodal electrode to cathodal one, it is referred to as transcranial direct current stimulation (tDCS). Otherwise, if the applied electrical current is sinusoidally changing in time, it is referred to as transcranial alternating current stimulation (tACS). So far, for treating DRE, tDCS has been preferentially used.

### General overview of tES and clinical settings in epilepsy

A classical generic tDCS device employs a battery powered controlled current generator connected to two large surface electrodes (one anode and one cathode, ~25–35 cm^2^ each) localized on the scalp. A constant current with a maximum output in the milliamp range (1–2 mA) is delivered through an electrode (cathode) over epileptogenic zone to hyperpolarize pyramidal cells in this region (inhibition) and the circuit is completed by an electrode (the anode) localized preferentially over a non-involved zone in epileptic patients.

**Figure 1:**
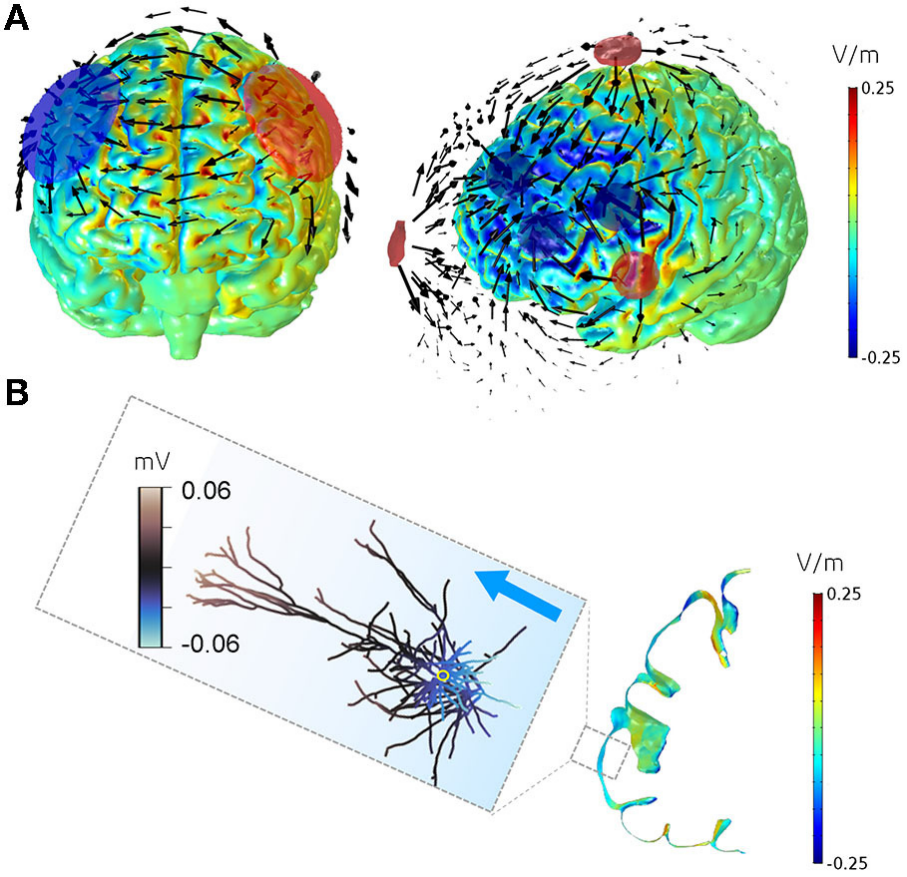
Simulation of tES-induced electric field on realistic brain model. **(A)** Electric field on a realistic brain model, generated by a bipolar montage with large sponge electrodes (left) and a multichannel montage employing six smaller electrodes (right). Cathodes are shown in blue, and anodes in red. Fields are shown for a total applied stimulation current of 1 mA for the bipolar and 4 mA for the multichannel montages. The colormap represents the component of the electric field directed perpendicularly to the cortical surface (or normal electric field, scale in V/m). The arrows display the current density field in the brain (not in scale). In our convention, the cortical normal vector points from the cortex surface to deeper brain regions, therefore outward currents give rise to negative values of the normal electric field component (in blue) and inward currents to positive values (in red). Normal electric fields are strongest under the electrode and in regions where the cortical gyration aligns the cortical column with the electric field between the electrodes. In multielectrode focal montages (right figure), the outward fields are particularly predominant under the cathodes. **(B)** Normal electric field and induced soma polarization for a total applied stimulation current of 1mA. Detail of the membrane polarization induced by an electric directed outwardly from the cortex on a realistic pyramidal neuron. The neuron morphology was reconstructed from Blue Brain Project. It is approximately located in the area in the dashed box, oriented perpendicularly to the cortex and exposed to an electric field value of 0.15 V/m along its axo-somatic axis and directed outwardly (blue arrow). The electric field causes a net polarization, or hyperpolarization, of the soma (located inside the yellow circle) of about−0.06mV from the resting potential.

The tES effects depend on several factors, including stimulation duration, current intensity, repetition, number of electrodes (bipolar or multichannel tDCS), and electrode position (Figure 1A).

The tES currents are associated with electric fields (EF) by a linear relation that depends on electrode positions, current (measured in mA), anatomy and conductivity (units of S/m). This is crucial, because the effects of tES are assumed to be mediated by the electrical fields in the cortex—with both intensity and direction being important (Ruffini et al., 2013). The electric field is vectorial, and its magnitude has units of V/m (or, equivalently, mV/m). It is a force field that induces ions to displace and accumulate across the membrane. The charge accumulation effect is larger in the dendritic-soma direction of elongated neurons, such as the pyramidal neurons (*in vitro* studies: Ghai et al., 2000; Lian et al., 2003; Bikson et al., 2004; Chang et al., 2015, or see Ruffini et al., 2014). The EF can be obtained by calculating the gradient of the voltage measured on each contact of a same intracranial stereoelectroencephalography (SEEG) electrode. Therefore, studies can estimate the amount of EF reaching the explored brain areas when using tES with a given current.

Since cortical pyramidal neurons are preferentially aligned in a normal direction to the cortical surface, the normal component of the electric field seems to be the most crucial for cortical modulation. If the current (and thus the electric field) are directed out of the cortex, as in the case of cathodal stimulation, the soma is made slightly hyperpolarized, i.e., less excitable (Figure 1B).

The direction of the electrical field components is loosely related to the nature of nearby electrodes, with cathodal electrodes predominantly inducing outward pointing (inhibitory) fields in the brain region underneath, although this is heavily influenced by the cortical folding (Figure 1A). Realistic head models can be used to analyze the electric fields associated to a montage (Miranda et al., 2013) and even to optimize them provided that a cortical target is at hand (Ruffini et al., 2014). In a clinical perspective, it is desirable to optimize stimulation focality and to prolong therapeutic after-effects. Thus, the understanding of underlying mechanisms could help us to determine the optimal parameters for tES in epilepsy. Although there is a growing body of research on tES, the stimulation parameters and the experimental designs are not consistent between studies and the resulting findings are at times contradictory.

The purpose of this systematic review is to provide an up-to-date summary of tES development in clinical epilepsy treatment and its effects on the underlying basic neurophysiology. Indeed, the neuro-modulatory mechanisms involved during the application of a weak current remain to be clarified.

## Materials and methods

### Review planning and searches

**Figure 2:**
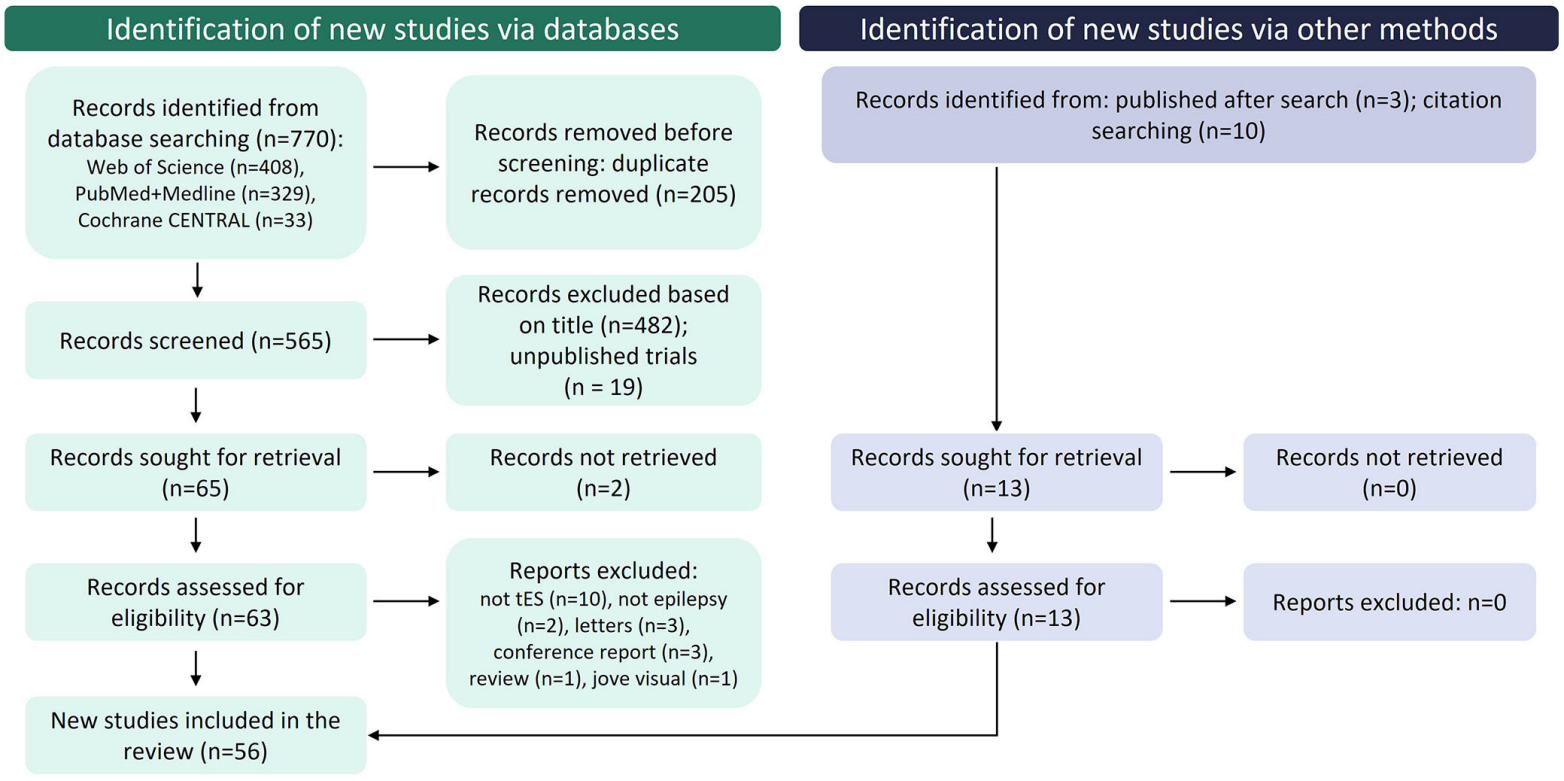
Flowchart of PRISMA record selection process.

This systematic review was performed in accordance with the Preferred Reporting Items for Systematic Reviews and Meta-Analyses (PRISMA) guidelines (Page et al., 2021). A database search was performed in November 2021 *via* the databases PUBMED, MEDLINE, Web of Science, and Cochrane CENTRAL by two independent reviewers. Articles whose title, abstract or keywords contained the keywords “epilepsy AND (transcranial current stimulation OR transcranial electrical stimulation)” were screened. Figure 2 shows the Prisma flowchart of the selection process.

### Screening and eligibility criteria

In the first place, duplicate records were excluded. After a screening based on title and abstract and performed by two independent reviewers, both clinical and fundamental research records regarding tES and epilepsy were submitted to eligibility. Articles not fulfilling the eligibility criteria (full-text records, original research concerning epilepsy and focusing on tES, English text available) were excluded. Selected articles are presented in the form of summary tables and of qualitative description of the main results described in the original studies.

## Results

### Retrieved papers

From a total of 770 records identified from the database search, 718 were excluded for several reasons, specified in the flowchart shown in Figure 2. A total of 49 studies fulfilled eligibility criteria and 13 studies were subsequently added through complementary methods. Among the included records, 23 were clinical studies with 17 clinical trials (Table 1) and 6 clinical cases (Table 2), 6 analyzed tES-related brain network changes and 27 focused on the physical and neurobiological effects of tES (9 records using animal models of epilepsy, 5 on epileptic patients, 7 *in vitro* and 6 studying computational models).

**Table 1:** Clinical trials with tDCS in epilepsy (*n* ≥ 5).

**References**	**Number of subjects**	**Epilepsy type**	**Methodology**	**Duration**	**Method outcome**	**Effects**	**Side effects**
Fregni et al. (2006)	19	Focal, malformation of cortical development (MCD)	Cathodal tDCS at 1 mA, 20 min (*n* = 10), sham (*n* = 9) Max follow-up: 1 month	1 session	EEG (21 electrodes), number of EDs, SF	Decrease of −64.3% of EDs −44% SF decrease at 1 month follow-up	Itching sensation
Varga et al. (2011)	5 (children)	Focal DRE, continuous spike-wave discharges during slow sleep (CSWS)	Small tDCS electrodes at 1 mA, 20 min before sleep + sham	2 sessions before sleep (1 active, 1 sham)	EEG (19 electrodes), EDs during slow sleep (spike-index)	Cathodal tDCS did not reduce EDs	No adverse event
Faria et al. (2012)	15 healthy subjects + 2 epileptic patients	DRE, CSWS	Heathy subjects: active 0.5 mA, 5 min + 1 mA, 5 min + sham Epileptic patients: 3 sessions (1 mA, 10 min during sleep for 3 days)	3 sessions	EEG (24 electrodes), EDs	Decrease of −45% EDs	93% of the participants did not feel anything
Auvichayapat et al. (2013)	36 (children)	Focal DRE	Cathodal tDCS at 1 mA, 20 min (*n* = 27), sham (*n* = 9) Max follow-up: 1 month	1 session	EEG (32 electrodes), EDs, SF, “Quality of life in childhood epilepsy questionnaire” (QOLCE)	Decrease of EDs (−45.3%) at 48 h, small decrease of SF at 1 month	1 patient had transient skin erythema
Tekturk et al. (2016a)	5	Rasmussen encephalitis	Cathodal and anodal tDCS: at 2 mA, 20 min + sham after 2 months	3 consecutive days	SF	>50% of decrease in SF	No adverse effects
Auvichayapat et al. (2016)	22 (children)	Lennox-Gastaut syndrome	Randomized double-mind-controlled Each day: active cathodal tDCS (*n* = 15) at 2 mA, 20 min, sham (*n* = 9) Follow-up: 4 weeks	5 consecutive days	EEG (32 electrodes), EDs, oxygen saturation	−8% decrease of ED at 1 month, more after 48 h, −55.9% decrease of SF	1 patient had superficial skin burn
Tekturk et al. (2016b)	12	Mesial temporal lobe epilepsy with hippocampal sclerosis (MTLEHS)	Cathodal tDCS at 2 mA, 30 min, + sham	3 days	SF	83% of responders (>50% of SF decrease)	Tingling sensation
Zoghi et al. (2016)	29	Focal temporal lobe epilepsy (TLE)	Cathodal tDCS active group (*n* = 20): 1 mA 9 min stim-20 min break-9 min stim + sham group (*n* = 9) Max follow-up 1 month	1 session	EEG, Paired-pulse transcranial magnetic stimulation: short interval intracortical inhibition (SICI) calculated from motor evoked potentials MEP, SF	Increase in SICI for experimental group compared to sham, Decrease of −42% of SF in active group	Itching, burning sensation, transient headache, neck pain
Assenza et al. (2017)	10	Focal TLE	Randomized controlled, Cathodal tDCS at 1 mA, 20 min + Sham	2 sessions (1 active, 1 sham)	EEG (19 electrodes), interictal epileptiform activity (EA)	No change in EA, decrease of SF: −71 ± 33% in active group vs. 25 ± 125% for sham	Itching sensation
San-Juan et al. (2017)	28	MTLEHS	Randomized placebo-controlled, double-blinded clinical trial Cathodal tDCS at 2 mA, 30 min for 3 days (*n* = 12) + Cathodal tDCS, 30 min for 5 days (*n* = 8) + Sham (*n* = 8) Max follow-up: 60 days	1 session	EEG, interictal epileptiform discharges (IEDs)	Decrease of IEDs immediately after tDCS, decrease in SF at 1 and 2 months after tDCS (3 days: −43%; 5 days: −55%) compared to baseline	Mild itching sensation, moderate headache
Karvigh et al. (2017)	10	Lateral frontal lobe epilepsy	HD-tDCS 2 mA, 20 min, no sham Max follow-up: 1 month	10 consecutive days	EEG (18 electrodes), EDs, SF, neurocognitive functions (neuropsychological tests)	Decrease of EDs (−35% for *n* = 5), increase of EDs (+48% for *n* = 5), decrease of 38.1% of SF at 1 month, improvement of attention and working memory after HD-tDCS	Mild headache
Lin et al. (2018)	9	Partial refractory epilepsy	2 mA, 20 min Max follow-up: 1 month	6 sessions of stim per month for 2 months	EEG (21 electrodes), EDs, Phase-lag-index (PLI), SF	No change in ED, decrease of PLI correlated to decrease of SF, Decrease of SF (−48 ± 31.2%) compared with baseline	Transient erythematous rash
Yang et al. (2019)	7	Epileptic spasms	Cathodal tDCS at 1 or 2 mA for 40 min (2 × 20 min with different electrode's positions) Follow-up: 28 days min, 4 months max	14 consecutive days of stim (for 1 or several times)	SF	Decrease in SF for short or long-term duration compared to the baseline (−46% at 4 months follow-up)	NA
Yang et al. (2020)	70	Focal DRE	Randomized, double-blind, sham-controlled, three-arm (Group 1: Sham, Group 2: 20 min stim, Group 3: 2 × 20 min stim), parallel multicentric study Cathodal tDCS at 2 mA Follow-up: 56 days	14 consecutive days	SF, QOLIE-31	Decrease in SF in Group 2 (−50.73–21.91%) compared to sham, decrease in SF in Group 3 (−63.19–49.79%) compared to sham Decrease in SFs in Group 3 (64.98–66.32%) compared with group 2 at 5 weeks follow-up No difference in QOLIE	Mild itching sensation
Kaufmann et al. (2021)	15	DRE	Cathodal tDCS at 2 mA for 9 min stim- 20 min break–9 min stim No follow-up	1 session	EEG (32–64-channels) IEDs, SF, Comfort Rating Questionnaire (CRQ)	Reduction in IEDs (−30.4 ± 21.1%), decrease of −48% in SF	Tingling feeling, burning sensation, slight tiredness
Kaye et al. (2021)	20	DRE	2-center, open-label study, personalized multichannel tDCS (max 8 electrodes) 10 sessions of 20 min of cathodal tDCS over 2 weeks Max follow-up: 8–12 weeks	10 sessions over 2 weeks	SF	SF reduction of −44% compared to baseline, 40% of responders	Increase in SF for 3 patients, tingling, itching sensation, transient dizziness, moderate and transient headache
Daoud et al. (2022)	10	Focal DRE	Personalized multichannel tDCS (max 8 electrodes) based on SEEG recordings, cathodal tDCS at 2 mA for 20 min stim- 20 min break–20 min stim Max follow-up: 6 months	3 sessions of 5 consecutive days, each separated by 2 months	EEG (19-channels), R2 strength, IEDs, SF	Decrease in R2 strength in responders in alpha and beta frequency bands, SF reduction of −48% compared to baseline at 2 months follow-up	Slight itching, slight dizziness during the stimulation

tDCS, Transcranial direct current stimulation; MCD, Malformation of cortical development; EEG, Electroencephalography; ED, Epileptiform discharges; CSWS, Continuous Spike-Wave Discharges During Slow Sleep; DRE, Drug-resistant epilepsy; SF, Seizure frequency; QOLCE, Quality of Life in Childhood Epilepsy Questionnaire; MTLEHS, Mesial temporal lobe epilepsy with hippocampal sclerosis; TLE, Temporal lobe epilepsy; SICI, Short interval intracortical inhibition; MEP, Motor evoked potentials; EA, Epileptiform activity; IEDs, Interictal epileptiform activities; HD-tDCS, High-definition tDCS; PLI, Phase-lag index; QOLIE-31, Quality of life in epilepsy.

**Table 2:** Clinical cases with tES in epilepsy.

**References**	**Number of subjects**	**Age**	**Epilepsy type**	**Age epilepsy onset**	**Seizure semiology**	**EZ**	**Methodology**	**Duration**	**Method outcome**	**Effects**	**Side effects**
Yook et al. (2011)	1 (F)	11	Focal cortical dysplasia	4 years	NA	Right temporo- parietal	Cathodal tDCS at 2 mA, 20 min, 5 times a week for 2 weeks Follow-up: 2 months	5 consecutive day for 2 weeks	Sleep EEG	Decrease of seizure duration Decrease of SF (-50%)	NA
San-Juan et al. (2011)	2 (2M)	P1: 31 P2: 17	Rasmussen encephalitis	P1: 28 P2: 15	P1: Generalized tonic-clonic seizures, epilepsia partialis continua, aphasia P2: Generalized tonic-clonic seizures, epilepsia partialis continua		P1: cathodal tDCS at 1 mA, 60 min in 4 sessions Follow-up: 12 months P2: cathodal tDCS at 2 mA, 60 min in 4 sessions Follow-up: 6 months	4 sessions	SF	P1: seizure free, improvement of attention P2: improvement of seizure intensity, improvement of motor functions, mild improvement of partial seizures	NA
San-Juan et al. (2016)	1 (F)	16	Pharmaco- resistant juvenile myoclonic epilepsy	12 years	Myoclonic absence	Anterior quadrant	tACS 1 mA at 3 Hz pulse train during 60 min Follow-up: 1-2 months	4 consecutive days	EEG, SF	75% increase of SF 15-day seizure free at 2 months follow-up	NA
Meiron et al. (2017)	1 (M)	30 months	Neonatal epileptic encephalopathy	5 days	Continuous tonic spasms, focal motor seizures, hemiconvulsions, generalized tonic-clonic seizures, focal myoclonus, suppression-burst pattern	Right temporal lobe	HD-tDCS 4 × 1-Ring configuration, 20 min each day for 10 days, at 0.1–1.0 mA	10 days	Video-EEG (32-channels), Paroxysmal epileptiform activity, hypsarrhythmic sharp waves, SF	No decrease in SF but lower sharp wave amplitude vs. baseline, vital signs, and blood chemistry stables	No adverse event
San-Juan et al. (2018)	1 (F)	28	Focal cortical dysplasia	9 years	Clonic and sensory right hemi-body seizures	Left frontal	2 mA for 30 min, long-term follow-up: 1 year	7 sessions over 9 weeks	EEG, counting spikes, SF	No change in the number of spikes, cumulative decrease in SF at long-term follow-up	Mild itching sensation
Marquardt et al. (2019)	1 (F)	15	POLG-related mitochondrial disease, multifocal epilepsia partialis continua	NA	Status epilepticus multifocal seizures with multiple semiology, epilepsia partialis continua	Right occipital region	2 mA for 20 min each day for 4 or 5 days	5 consecutive days	EEG (25- channels), spike frequency, jerk frequency	No change in jerk frequency and spike	NA

tDCS, Transcranial direct current stimulation; EEG, Electroencephalography; SF, Seizure frequency; HD-tDCS, High-definition tDCS; tACS, Transcranial alternative current stimulation.

### Clinical studies

#### Seizure frequency

Cathodal tDCS has been used in several clinical studies with pediatric and adult patients suffering from DRE. Table 1 shows a summary of clinical trials of patients (*n* ≥ 5 patients) with epilepsy treated with cathodal tDCS and Table 2 summarizes the clinical cases (*n* < 5) using tDCS for treating epilepsy.

The first controlled clinical trial using cathodal tDCS on patients suffering from refractory epilepsy was conducted by Fregni et al. in 2006 (Fregni et al., 2006). This study investigated the effects of one cathodal tDCS active session stimulation of 20 min at 1 mA in 19 patients with DRE compared to sham stimulation. They showed that cathodal direct-current polarization decreased seizure frequency (mean decrease of −44%) compared to the baseline. Most studies are consistent with these results, reporting positive effects of cathodal tDCS in epileptic patients reducing seizure frequency (SF). Indeed, focusing on SF change due to cathodal tDCS, 15 clinical trials (15/17) reported a decrease or a trend to a decrease in SF. Auvichayapat et al. (2013) reported a slight decrease in SF 1 month after one session of 20 min of cathodal tDCS at 1 mA in children suffering from DRE (*n* = 36 children, 27 with active stimulation and 9 with sham stimulation), highlighting the safety of this technique for young population and its clinical usefulness. Two other studies (San-Juan et al., 2011; Tekturk et al., 2016a) reported significant (>50%) decrease in SF in patients suffering from Rasmussen's encephalitis, a rare and progressive inflammatory disease that reaches one cerebral hemisphere, leading to intractable partial-onset seizures. Another clinical pediatric trial conducted by Auvichayapat et al. (2016) has shown reduction in SF of 55.9% in patients with epileptic spasms and Lennox Gastaut syndrome (LGS) compared to sham group 1 month after 5 consecutive days of 20 min tDCS at 2 mA. Then, 28 patients suffering from mesial temporal lobe epilepsy with hippocampal sclerosis have been enrolled in a randomized placebo-controlled, double-blinded clinical trial where they received one session of tDCS at 2 mA for 3 or 5 days (San-Juan et al., 2017). Two months after the cathodal tDCS session, they obtained a decrease of −43% in SF for the group with 3 days of stimulation and a decrease of −55% in SF for the group receiving tDCS for 5 days compared to baseline. Thus, the heterogeneity of epilepsy types among studies demonstrates the potential efficacy of cathodal tDCS for treating several etiologies of refractory focal epilepsy.

The tDCS technique has been improved over the last few years with the recent use of several pairs of smaller electrodes (multichannel tDCS), contrasting with conventional tDCS using two large rectangular electrodes (see Figure 1A). Indeed, conventional tDCS, using one electrode (cathode) placed over the epileptogenic region and one anodal electrode placed on the scalp over a non-involved zone, allowed a modulation of cortical activity in a relatively larger area than that covered by the target electrode (Nitsche et al., 2007). In addition to more precise targeting of the generated electric field over the desired area, an enhanced stimulation focality would avoid unwanted excitation of non-target areas. Thus, neurophysiological and modeling studies have shown that smaller multiple electrodes produce more targeted outcomes and that their placement is crucial for the effects of stimulation (Ruffini et al., 2014; Hannah et al., 2019). This focal method has been previously used in the clinical field with encouraging results. A recent study confirmed the safety of multichannel tDCS applying 20 min of stimulation (4-Ring configuration) each day for 10 days in a 30-month-old child suffering from neonatal epileptic encephalopathy (Meiron et al., 2017). Then, a clinical trial enrolling 10 patients with intractable lateral frontal lobe epilepsy has shown changes in SF with a decrease of −38.1% 1 month after the multichannel tDCS at 2 mA applied for 10 consecutive days (Karvigh et al., 2017). Another interest of using multifocal tDCS is to use biophysical modeling and electrophysiological or imaging data to optimize electrode montage to target and avoid specific brain regions depending on their degree of epileptogenicity. Recently, an open label study has been conducted on epileptic pediatric population using personalized multichannel tDCS (max 8 electrodes) for 10 sessions of 20 min of cathodal tDCS over 2 weeks (Kaye et al., 2021). Multi-electrode montages were designed using a realistic head model-driven approach to apply an inhibitory electric field to the target cortical seizure foci and surrounding cortex to suppress excitability and reduce seizure rate. They demonstrated the feasibility and the efficacy of a customized multichannel cathodal tDCS as antiepileptic protocol with a −44% seizure reduction and a responder rate of 40%.

If the clinical benefit of tES is further demonstrated, one of the main objectives of future studies will be to prolong the positive after-effects on SF. In this regard, performing several tDCS sessions is expected to decrease SF in the long-term. Indeed, previous studies only performed one tES session with promising short-term results whereas a clinical case conducted by Yook et al. highlighted that the repetition of stimulation allows to prolong and enhance the effects of tDCS. They showed a decrease in SF lasting for 2 months after 2 consecutive weeks of daily stimulations (Yook et al., 2011). San-Juan et al. (2017) observed greater clinical improvement in patients with mesial temporal lobe epilepsy after 5 consecutive days of tDCS than those who received the treatment for 3 days. Interestingly, the duration of after-effects is dependent on the length of the interval between stimulation sessions. Thus, Monte-Silva et al. (2010) explored the effects of different break durations between cathodal tDCS stimulation sessions in healthy subjects. They showed that the magnitude of cathodal tDCS-induced inhibition was enhanced after a break interval of 20 min between two 9 min stimulation sessions but not after a 3 min break. This specific timing effect seems to be important for optimizing cumulative effects due to tDCS. Further recent studies also tried to lengthen tDCS after-effect modifying the interval duration. Recently, a multicentric controlled study conducted by Yang et al. in 2020 showed a significantly greater reduction in SF (64.98–66.32%) in the group of epileptic patients receiving 2 × 20 min of stimulation with 20 min off compared to the group receiving only 20 min of stimulation at 5 weeks follow-up (Yang et al., 2020). Then, another clinical trial performing 2 × 9 min stimulation with 20 min break at 2 mA in epileptic patients has shown a mean decrease of SF about 48% (*n* = 15) immediately after one session of cathodal tDCS (Kaufmann et al., 2021).

Regarding the main limitations of previous clinical studies, we noticed a lack of data regarding a potential improvement of quality of life and of seizure severity after tES, as the majority of studies focused on seizure frequency only.

Furthermore, the most common limit of previous studies is the lack of long-term follow-up, the small sample size of patients and the large and heterogeneous epilepsy types. To date, only one randomized, double-blind, sham-controlled, multicentric study on tES in epilepsy has been conducted (Yang et al., 2020), including 70 patients suffering from DRE and divided into three groups: group 1 with sham stimulation, group 2 with 20 min of stimulation and group 3 with 2 × 20 min stim at 2 mA during 14 consecutive days. They obtained a significant decrease in SF in active groups compared to sham group, with better results for group 3 compared to group 2 at 8 weeks follow-up. They reported a decrease in SF in group 2 of −50% the first 4-weeks of follow-up compared to baseline and a decrease of −25% in SF the second 4-weeks of follow-up. The group 3 obtained also a −50% decrease in SF in the first 4-weeks of follow-up but a decrease of −45% in SF after the second 4-weeks of follow-up. Regarding the sham group, they observed a decrease in SF in group of −25% the first 4-weeks of follow-up compared to baseline and a decrease of −12.5% in SF the second 4-weeks of follow-up. Thus, the beneficial effects lasted longer when the patients received 2 × 20 min of tDCS. However, they did not find any difference in quality-of-life scale (QOLIE) after tDCS treatment.

#### Epileptiform discharges

Studying clinical outcomes, one of the main techniques to quantitatively measure the benefit of tES in epileptic patients is focusing on change in the count of interictal epileptiform discharges (IEDs) using electrophysiological recordings.

Among clinical studies, 6 clinical trials reported a decrease in interictal epileptic discharges using scalp EEG recordings (Fregni et al., 2006; Faria et al., 2012; Auvichayapat et al., 2013, 2016; San-Juan et al., 2017; Kaufmann et al., 2021). First, Fregni et al. showed significant reduction in IEDs (64.3%) in epileptic patients receiving the active tDCS treatment (*n* = 10) compared to the sham group (*n* = 9) (Fregni et al., 2006). Then, Auvichayapat et al. (2013) reported improvement in EEG abnormalities in 36 children suffering from intractable focal epilepsy (reduction in IEDs of 45.3% for 48 h) after one active stimulation session of cathodal tDCS at 1 mA for 20 min (*n* = 27) compared to sham group (*n* = 9).

In contrast to these findings, 3 clinical trials (Varga et al., 2011; Assenza et al., 2017; Lin et al., 2018) and two clinical cases (San-Juan et al., 2018; Marquardt et al., 2019) reported no effect of cathodal tDCS on IEDs and one clinical study revealed an increase of 48% in interictal epileptiform discharges after cathodal tDCS (Karvigh et al., 2017). Indeed, in a pediatric clinical study conducted by Varga et al. (2011) in five children suffering from refractory continuous spike and waves during slow sleep, they found no reduction of spike-index and they assigned this result to the use of smaller tDCS electrodes than those usually used in conventional tDCS.

Furthermore, focusing on multichannel tDCS studies, Karvigh et al. (2017) reported a change in IEDs after repeated multichannel tDCS in epileptic patients. However, the change of IEDs was different between patients. They indeed reported an increase in IEDs in five patients, while IEDs had decreased in the five others immediately and 1 month after high-definition tDCS (HD-tDCS).

Thus, several clinical trials of tES in epilepsy did not report information regarding potential change in IEDs (Tekturk et al., 2016a,b; Yang et al., 2019, 2020; Kaye et al., 2021).

These discrepancies may come from the variability of study designs, stimulation protocols and parameters such as the current intensity, stimulation duration, repetition of sessions, interval between sessions, electrode size, number, and localization of electrodes on the scalp. Further studies should standardize the stimulation parameters in order to optimize the clinical benefit of this technique by being more reproducible and efficient in epilepsy therapy.

Finally, despite the vast number of studies investigating the effects of tACS in memory, sleep and other functions, there is little account for tACS effects on epilepsy. One study conducted by Holmes et al. (2019) implemented a slow-pulsed tACS protocol in seven adult DRE patients with MRI-based personalized head models. The study consisted of 5 consecutive days of tES protocol combined with 256-channel dense EEG recordings before and after stimulation. Each tES pulse measured 100 ms in duration, with a 0.5 Hz stimulation frequency. They reported spike suppression without worsening of epileptiform activity, highlighting the safety and the efficacy of tACS to suppress IEDs. The use of IEDs as a quantifying marker for tES studies is a matter of debate. Indeed, since the presence of IEDs in scalp EEG recordings is not systematic and is not always related to seizure risk, the use of alternative electrophysiological methods for quantifying tES effects on the brain should be developed and explored in further trials.

#### Safety

Cathodal tES is a safe, non-invasive method of neuromodulation showing only mild adverse events and transient side effects. To date, no serious adverse event has been reported in studies using cathodal tDCS as epilepsy treatment. The more common effects are slight itching (Fregni et al., 2006; Zoghi et al., 2016; Assenza et al., 2017; San-Juan et al., 2017, 2018; Yang et al., 2020; Kaye et al., 2021), tingling (Tekturk et al., 2016b; Kaufmann et al., 2021; Kaye et al., 2021), mild skin erythema (Faria et al., 2012), and less frequently transient and moderate headache post-session (Zoghi et al., 2016; Karvigh et al., 2017; San-Juan et al., 2017; Kaye et al., 2021), slight tiredness (Kaufmann et al., 2021) and burning sensation during the current application (Auvichayapat et al., 2016; Zoghi et al., 2016). Numerous studies reported no side effects at all (San-Juan et al., 2011; Varga et al., 2011; Faria et al., 2012; Tekturk et al., 2016a; Meiron et al., 2017). Kaye et al. (2021) reported that three participants in their study experienced an increase in seizure frequency (>50%) during treatment. In one participant, the cause might have been the presence of several undetected seizure foci. Their results suggest that high certainty regarding the epileptic focus for appropriate targeting is desirable and that stimulation should be stopped immediately if convincing evidence of a worsening of seizures is seen. Their data also suggested that if stimulation is stopped, a return to the baseline seizure frequency will occur.

The use of tACS in epilepsy is still limited. As for tDCS, previous studies using tACS only reported minor side effects. The safety and effects of tACS have been assessed in 13 DRE patients undergoing stereo-EEG investigation during NREM sleep and waking rest by Lafon et al. (2017). Sinusoidal tES was applied at either 0.75 Hz or 1 Hz with standard stimulation intensities (up to 2.5 mA; maximum induced field: 0.43 V/m). Although tACS failed to entrain spindle, gamma, or theta activity during neither NREM sleep nor waking rest, this study has proven the safety of low frequency tACS in epilepsy patients implanted with intracranial electrodes. Moreover, the tACS safety has also been proven with simultaneous tACS-SEEG recordings in both animal and human studies. Opitz et al. (2016) investigated the electric field distribution in non-human primates and human epileptic patients during tACS and tDCS with variable frequencies and current intensities. Thus, they positively assessed the safety and the possibility of reaching subcortical structures.

### Network effects of application of weak electric current in epilepsy

The human brain is characterized by specific structural and functional interconnection patterns linking cortical regions on short or long-distance. Neurological diseases inducing changes in synaptic plasticity may affect the communication within and between neuronal populations (Bettus et al., 2008; Warren et al., 2010; Varotto et al., 2012; Van Diessen et al., 2013; Lagarde et al., 2018). Critical features of pathological networks can be captured by measuring and localizing brain segregation and integration processes. Functional connectivity (FC) is typically used to define statistical temporal dependencies among neuronal signals measured from different brain regions. Thus, several methods (both linear and non-linear) have been developed for estimating FC. Synchrony of signals obtained from imaging and electrophysiological studies allow to characterize FC between different, distant brain regions.

Previous investigations have provided increasing evidence that epilepsy is a large-scale brain network disorder, not limited to a focal epileptogenic area (Pittau and Vulliemoz, 2015; Bartolomei et al., 2017). Thus, even focal drug-resistant epilepsies are associated with interictal alteration in FC involving the nodes of the epileptogenic network. Using EEG, SEEG, magnetoencephalography (MEG), and functional magnetic resonance imaging (fMRI) data, previous studies have shown that the functional disturbances are characterized by reinforced FC within epileptic cortices [epileptogenic zone (EZ) and propagation zone (PZ)] compared to the non-involved zone (NIZ) (Bettus et al., 2008; Warren et al., 2010; Van Diessen et al., 2013; Bartolomei et al., 2017), whereas the connectivity between EZ and the NIZ is lower. These studies also showed that an increased FC within the NIZ is associated with a poorer post-surgical outcome. The reduced synchrony observed between EZ and the surrounding NIZ indicates that the EZ may be functionally disconnected from surrounding brain regions (Warren et al., 2010).

Observations of enhanced connectivity within the EZ suggested that this disturbance within the epileptogenic network may facilitates seizure occurrence and propagation by generating the exacerbated synchronization during seizures. This hypothesis defines new pathways for treatment. Thus, it seems reasonable to hypothesize that an efficient antiepileptic therapy would restore the normal FC decreasing abnormal coupling within EZ and between EZ and PZ. Recent studies indeed demonstrated that global brain network dynamics are linked to clinical outcome of pharmacological antiepileptic treatments and that such epilepsy-related FC changes are reversible and can be controlled by AEDs (Clemens et al., 2014; Anderson et al., 2020). As far as neurostimulation is concerned, clinical studies using invasive neuromodulatory devices, such as vagus nerve stimulation (VNS) have shown a decreased synchrony induced by VNS as anti-epileptic mechanism in patients with good response (Fraschini et al., 2013; Bodin et al., 2015; Sangare et al., 2020).

Recent clinical studies applying tES on epilepsy patients have as well found tES-induced FC modifications, which could accompany the decrease in epilepsy features after tES. In this section, we analyze the recent findings on tES-induced network changes in epilepsy patients and their relation to the clinical outcome of stimulation. Stimulation *via* tDCS can modulate the whole brain networks, rather than just the stimulated cortical area localized under the scalp electrode (Luft et al., 2014). Indeed, some studies performing cathodal tDCS on healthy participants have been conducted. Polanía et al. investigated the tDCS-induced effects on cortical network function using high resolution EEG (62 channels). They found interhemispheric and interhemispheric connectivity changes in several frequency bands (4–90 Hz) after excitatory anodal tDCS over the primary motor cortex (Polanía et al., 2011). Then, in a simultaneous high definition tDCS-EEG study, they reported that cathodal stimulation had induced significant changes in global broadband cortical activity compared to sham stimulation (Roy et al., 2014). Applying cathodal stimulation resulted in lower global synchronization across frequency bands compared to sham and anodal stimulation, interpreted as an inhibitory action of cathodal stimulation.

Furthermore, in the clinical field, a recent double-blind sham-controlled study focused on FC changes after tDCS in epileptic patients has been performed (Tecchio et al., 2018). This study aimed to explain part of cathodal tDCS clinical effects in six DRE patients with FC changes. Both cathodal tDCS and sham stimulations have been performed in each patient at 1 mA for 20 min for the active stimulation. They reported that the decrease of SF due to tDCS was correlated with an increase of coherence measures in epileptogenic focus in the whole frequency band and in theta band, compared with sham stimulation. Thus, the clinical improvement of epileptic patients was correlated with functional changes of EZ. On the contrary, another clinical trial, conducted by Lin et al. (2018), investigated the effects of repeated sessions of cathodal tDCS in nine patients suffering from partial refractory epilepsy. Here, they estimated FC changes studying modifications of phase-lag-index (PLI) and they obtained a negative correlation of PLI in alpha band and the SF decrease-induced by tDCS. They considered that tDCS induced a cumulative clinical benefit with reduction in SF in epileptic patients, associated with a decrease in synchronization. A more recent study aimed to evaluate and predict the potential clinical efficacy of cathodal tDCS by analyzing the induced functional network alterations (Hao et al., 2021). The 27 epileptic patients were separated in two groups of stimulation: sham group and active cathodal tDCS group with 20-min stimulation at −1 mA, for 5 consecutive days, targeting the regions with higher IEDs rate. Graph theoretical analysis has been based on fMRI data performed before the tDCS treatment and after the 5-days stimulation. They reported tDCS-induced functional alterations with a significant decrease of graph theoretical measures only for the patients presenting a good response to tDCS, acknowledging the potential of fMRI-based graph theoretical measures for clinical prediction of tDCS outcome in epileptic patients. Furthermore, using EEG data, another controlled tDCS trial conducted on 25 DRE patients has shown an attenuation of the functional network's connectivity (Luo et al., 2021). Indeed, they estimated changes in FC between brain networks using graph-theory metrics such as average clustering coefficient, characteristic path length and small-world index calculated from scalp EEG recorded at baseline, at the end of 5-days cathodal tDCS at −1 mA for 20 min, and at 4 weeks follow-up. They observed a decrease in neural transfer efficiency after cathodal tDCS and a slight increase in characteristic path length. The measure of characteristic path-length is controversial in most studies in epilepsy. Thus, they hypothesized that the increase in path length may be explained by the loss of connectivity between the nodes, and that the change in small-world index after cathodal tDCS is more meaningful to reflect functional induced changes. In this study, the alterations in FC were only associated with significant IED reduction and may be responsible for the electrophysiological protection of the brain limiting seizure onset and seizure propagation. Recently, we have studied the link between EEG functional connectivity changes and the response to multichannel tDCS (Daoud et al., 2022). This study included 10 drug resistant epilepsy patients. Multichannel tDCS was applied during three cycles (one cycle every 2 months) of stimulation. Each cycle consisted of 5 consecutive days where patients received tDCS daily in two 20 min sessions separated by 20 min. After the last tDCS session, five patients experienced a SF decrease of 50% or more compared with baseline (R: responders). FC changes between cycles and across R and non-responder (NR) patients was estimated using linear correlation. R presented a significant decrease in FC at the third session in alpha and beta frequency bands.

To conclude, since some studies suggested that cathodal tDCS showed long-term cumulative effects of functional disturbances, a possible mechanism underlying this electrophysiological plasticity focused on network's changes might be explained by inhibition and long-term depression (LTD). Consequently, cellular, and molecular studies are needed to shed light on the involved underlying mechanisms due to tES. Thus, *in vivo, in vitro*, and computational studies are needed to address this gap in knowledge.

### Neurobiological aspects

The inter-subjects and inter-studies variability in clinical efficacy and in network changes underlies the need for a better identification of the basic mechanisms of tES. To discover the underpinnings of tES on brain activity, external weak electrical fields have been applied to *in vitro, in vivo*, and *in silico* (computational) models. Moreover, to identify the actual current flow inside the brain during tES and evaluate its potential to reach deep brain structures, various studies have taken advantage of intracranial recordings from DRE patients during tES stimulation (Opitz et al., 2016; Huang et al., 2017; Louviot et al., 2022). Here, we present the main findings on tES modulation of epileptic activity from *in vitro, in vivo, in silico* and human models.

Since the objective of this review is to summarize the findings on the use of tES in epilepsy, we will not present studies on rhythm entrainment on normal brain activity. For comprehensive reviews on such topics, the reader is referred to Reato et al. (2013) and Liu et al. (2018).

#### *In vitro* studies

*In vitro* studies have been useful to observe the cellular effects of tES *via* the application of either alternating (AC) or direct current (DC) electric fields (EF) on brain slices. The most common setup consists in placing two parallel Ag-AgCl plates or wires in the bath containing hippocampal or cortical slices and applying a certain current across the slice. Therefore, it is possible to record both intracellularly and extracellularly. To reproduce epilepsy *in vitro* (creating hyperexcitability), the standard artificial cerebrospinal fluid bath is modified by adding or reducing specific components. The main models use either bicuculline, 4-aminopyridine, picrotoxin, low-Ca^2+^ or high-K^+^ concentration changes to induce epileptiform activity. To offer a clearer overview of the used methods, Table 3 summarizes the epilepsy model, stimulation methods and electric field intensities of all *in vitro* studies here included.

**Table 3:** Models and stimulation methods used in *in vitro* studies.

**References**	**Model**	**AC/DC**	**Stimulation**	**EF magnitude**
Ghai et al. (2000)	Low-Ca2+ rat model of epilepsy *in vitro*, hpc CA1 region brain slices	DC only	2 parallel AgCl-coated wires placed on the surface of the ACSF in the interface chamber. Changes in EF orientation angles.	1–5 V/m, min 3.7 V/m for suppression activity
Bikson et al. (2001)	Rat hpc CA1 and CA3 brain slices, 3 different ACSF models of spontaneous bursting (low Ca, high K) and picrotoxin	AC only	AgCl-coated wires on surface of ACSF, square biphasic wave or 50 Hz sin wave + 20,50,500 and 5,000 Hz stim only in spontaneous low-Ca2+ bursting	Min ±25 V/m, Max ±200 V/m
Lian et al. (2003)	Rat hpc CA1 or CA3 brain slices, 3 different ACSF models of spontaneous bursting (low Ca, high K) and picrotoxin	DC and AC	Same as above (uniform external EF) + local monopolar stimulation to mimic DBS, 50 Hz	±160 V/m
Bikson et al. (2004)	Rat hpc slices, intracellular and voltage-sensitive dye recordings	DC and AC	AgCl wires >40 mm long, placed >15 mm apart or sintered Ag–AgCl cylindrical pellet electrodes 12 mm long and placed >5 mm apart (for stronger fields)	Min ±40 V/m
Chang et al. (2015)	Mouse thalamocingulate slices, 4-aminopyridine and bicuculline.	DC only	2 V/m cathodal DC + higher EF magnitude (max 16 V/m) to induce an immediate effect on seizure-like activity. For LTD analysis: 15 min of 4 V/m cathodal DC stimulation applied after 15 min of baseline	2–16 V/m
Rahman et al. (2017)	Rat primary motor cortex slices; anodal DC stimulation: EF > 0 on M1, opposite for cathodal	DC only	15 (adaptation) or 200 (post-adaptation) constant-current pulses (0.2 ms, 10-150 uA) or uniform extracellular EFs. Pre-synaptic afferent axons were stimulated at 5, 10, 20, and 40 Hz to simulate synaptic activity with c-DCS or a-DCS	±10 and ±20 V/m
Sun et al. (2020)	*In vitro*: mouse M1 and human post-operative neocortex slices, *in vivo*: mouse kainic acid (KA)-seizure model. Drug: NMDAR blockers (memantine/D-AP5)	DC only	cDCS: 400 μA, 25 min; cathode: proximal to the cortical pial surface, anode: beneath the subcortical WM.	9.2 V/m in the ACSF; 2.3 V/m at the recording surface

AC, Alternating Current; DC, Direct Current; hpc, hippocampus; CA1, CA3, hippocampus Cornu Ammonis 1 and 3; Ca^2+^, calcium; K^+^, potassium; ACSF, artificial cerebrospinal fluid; Ag, silver; AgCl, silver chloride; M1, primary motor cortex; LTD, long term depression.

Most *in vitro* studies employed DC fields to demonstrate its ability in controlling epileptogenic biomarkers and seizure features. Applied cathodal EFs show suppression of burst activity in hippocampal (Ghai et al., 2000; Lian et al., 2003; Bikson et al., 2004) and thalamocingulate slices (Chang et al., 2015). The efficacy of DC fields in acute suppression of epileptiform-like activity depends on EF polarity (Ghai et al., 2000; Lian et al., 2003; Bikson et al., 2004; Chang et al., 2015). Chang et al. (2015) demonstrated how cathodal DC stimulation, if oriented parallel to the dendritic-somatic axis, can control seizure duration and propagation in slices of mice anterior cingulate cortex. Ghai et al. (2000) demonstrated that cathodal DC application suppresses bursts more efficiently when they are parallel to the cell dendritic-somatic axis. Moreover, they showed how the *in vitro* low-Ca^2+^ model of epilepsy requires lower EF intensities than those required for epileptiform activity suppression in the low-K^+^ model. Such finding could be due to the induced cell swelling of the low-Ca^2+^ cellular environment, which in turn leads to reduced extracellular volume and higher tissue resistance. Thus, DC fields efficacy depends not only on the cellular orientation with respect to the EF, but also on the model of epilepsy and osmolarity. A possible limitation to the use of DC fields is the post-stimulation rebound that was shown to occur even after short DC pulses (Lian et al., 2003). Nevertheless, this rebound was caused by an electric field magnitude of up to 160 V/m, which is unrealistically obtained in human brains during tES (where, from both measurements and models we know that EF intensity is of the order of 1 V/m) and thus should not have an impact on clinical tES protocols. Moreover, DC fields do not modulate all cortical layers uniformly, which might be the reason for the variable cathodal-tDCS clinical efficacy (Sun et al., 2020).

Cathodal DC fields show both short- and long-term effects on epileptic-like activity, which would be supported, respectively, by neuronal hyperpolarization (Ghai et al., 2000) and long-term depression (LTD)—like mechanisms (Lian et al., 2003; Chang et al., 2015). Regarding long-term effects of stimulation, cathodal tDCS (cathodal tDCS) was shown to induce post-synaptic depression even after 15 min of stimulation, but only when combined with background spontaneous synaptic activity (Chang et al., 2015). In this study, such effects did not apply to the pre-synaptic currents and lasted more than 1 h, suggesting the involvement of LTD mechanisms. Indeed, Chang et al. demonstrated that the application of the N-methyl-D aspartate (NMDA) receptor antagonist abolished tDCS-induced LTD, highlighting the fundamental role of NMDA-mediated plasticity in tES long-term effects. Nevertheless, contradicting results of NMDA's role in long-term DC modulation comes from Sun et al. (2020). In this study, the authors demonstrate how cathodal DC stimulation coupled with NMDA receptor block produces LTD in different cortical layers, both in mouse primary motor cortex and human neocortex *in vitro* slices. Such contradicting findings may be due to differences in brain slices (thalamocingulate in Chang et al., motor cortex and neocortex in Sun et al.) or type of stimulation paradigm (see Table 3 for details on the stimulation parameters).

Although less investigated than DC fields, AC stimulation has been studied in various *in vitro* studies. Like DC fields, AC stimulation can suppress burst activity in epilepsy models (Bikson et al., 2001; Lian et al., 2003). Differently from DC stimulation, AC stimulation-induced polarization does not depend on orientation (Bikson et al., 2001; Lian et al., 2003) and does not show any post-stimulation rebound (Lian et al., 2003). Instead, AC stimulation induces different effects depending on the stimulus frequency. Bikson et al. (2001) showed that AC stimulation at 500 and 5,000 Hz could not suppress burst activity at any field strength, while 20 and 50 Hz had very similar effects and significantly reduced epileptiform discharges. In the same study, they further showed that neither stimulus waveform nor cell morphology influenced the EF-induced polarization. Interestingly, Bikson et al. (2004) reported the first direct measurement of membrane time constant (ranging from 14 to 70 ms), crucial for understanding the induced polarization of external EF. This result indicates that neurons should be less sensitive to relatively fast AC electric fields (>15 Hz) because of the quite slow polarization recovery of the membrane.

*In vitro* studies are particularly suited for understanding the mechanisms underlying the effects of electric fields on brain tissue, as induced by tES stimulation. Concerning DC fields, studies have shown that stimulation induces linear polarization in neurons. For EF magnitudes below 40 V/m, every 1 V/m of stimulation intensity will typically induce a membrane polarization of about 0.12 V/m in pyramidal cells (Bikson et al., 2004). While the underlying mechanisms of DC fields in epileptiform bursts suppression appear to be represented by membrane hyperpolarization (Ghai et al., 2000; Rahman et al., 2017), the mechanisms underlying AC effects would be more complex (Bikson et al., 2001; Lian et al., 2003). Indeed, AC stimulation induces an increase (2.5 mM ± 0.5, *n* = 5) in extracellular K+ concentration lasting for the whole stimulus duration and related to burst suppression (Lian et al., 2003). These authors moreover showed that bursts started again after a decrease in K+ extracellular concentration.

It is nevertheless important to notice how the above-mentioned studies were carried out with EF intensities significantly stronger than the standard intensities observed intracranially in tES human studies. It is estimated that a tDCS intensity of 1 mA generates electrical fields at the level of cortical neurons in the 0.2–0.5 V/m range (Datta et al., 2009; Miranda et al., 2013), while 2 mA produce an EF of about of 1 V/m (the reader is referred to the review summarizing studies non-involving epilepsy models such as Jackson et al., 2016; Vöröslakos et al., 2018). Indeed, only Ghai et al. (2000) and Chang et al. (2015) use EF intensities ranging 2–16 V/m, which are nevertheless at least twice higher than the EF produced by a 2 mA tDCS. Therefore, more efforts should be made to characterize the translation of *in vitro* studies to *in vivo* ones, evaluating the reliability of seizure models, quantifying the differences between thin slices and real brains with gyri and sulci (the EF is uniformly distributed in slices, but not uniformly distributed in real brains), and adapting EF intensities to the ones realistically used in human studies. *In vivo* studies are pivotal for implementing more realistic stimulation parameters within *in vitro* investigations.

#### *In vivo* studies

Animal models of epilepsy (depicting epilepsy features such as spike and wave discharges, generalized epilepsy-like seizures, focal epilepsy, status epilepticus models) can be genetic or obtained from various electrical or chemical procedures. For a comprehensive overview on these models, not subject of the current review, we refer the reader to Pitkänen et al. (2017). All records included in this review used rat models of epilepsy. More precisely, the used models consisted in: cortical-ramp stimulation to induce focal epilepsy (1 record, Liebetanz et al., 2006); spontaneous recurring spike-wave patterns (2 records, Berényi et al., 2012; Kozák and Berényi, 2017); WAG/Rij genetically modified rats (1 record, Zobeiri and van Luijtelaar, 2013) as model of absence epilepsy; lithium-pilocarpine (1 record, Kamida et al., 2011) and kainic acid (1 record: Wu et al., 2020) as status epilepticus models; pentylenetetrazol (2 records: Dhamne et al., 2015; Regner et al., 2020) as a model of either acute or chronic generalized seizures.

Cathodal tDCS efficiently increased the threshold of seizure triggering and latency of the first seizure after epilepsy induction (Liebetanz et al., 2006; Dhamne et al., 2015; Regner et al., 2020; Wu et al., 2020). Other outcomes concerned decrease in seizure duration, frequency, and severity (Kamida et al., 2011; Dhamne et al., 2015; Wu et al., 2020). In addition, cathodal tDCS led to changes in epileptiform discharges such as decrease of number and duration of spike-and-wave complexes (Zobeiri and van Luijtelaar, 2013; Wu et al., 2020), and reduction of epileptic bursts (Dhamne et al., 2015). Despite the positive results in seizure rate and epileptiform discharges reduction when applied interictally, tES does not seem to be able to stop ongoing seizures (Dhamne et al., 2015). Nevertheless, some studies have shown the potential of tES combined with pharmacological treatment, demonstrating that adding either lorazepam (Dhamne et al., 2015) or diazepam (Regner et al., 2020) to the tDCS stimulation can efficiently increase the antiepileptic effects of stimulation (abortion of seizures, threshold increase for the first seizure). Therefore, association of tDCS with pharmacological treatment may potentially reduce drug doses and thus, the drug's side effects.

Moreover, cathodal tDCS induces changes of signal frequency content: during stimulation, power in delta band was increased and power in alpha, beta and theta bands decreased (Zobeiri and van Luijtelaar, 2013; Dhamne et al., 2015). Wu et al. (2020) found that applying cathodal tDCS before status epilepticus induction *via* kainic acid injections led to a small increase of low frequency power (3–6 Hz) and an important decrease of high frequency content (35–37 Hz) in the cathodal tDCS treated group with respect to sham. In the study, they show that the high frequency content is associated with epileptic polyspikes, while the low frequency content would represent spike and wave patterns. Therefore, frequency power changes would indicate that tDCS inhibits neuronal networks by the inhibition of polyspike initiation, while shifting the tendency toward spike and wave patterns. Consistently with *in vitro* and human studies, *in vivo* stimulation effects are intensity dependent. More specifically, whereas a 100 μA current can cause a reduction in number of spike-and-wave patterns, only a 150 μA current could affect the duration of spike and wave episodes during cathodal tDCS and for about 1 h after stimulation (Zobeiri and van Luijtelaar, 2013). Similarly, Liebetanz et al. (2006) showed that, whereas 30 min of cathodal tDCS at 100 μA had no effect on the seizure threshold, a stimulation of 60 min led to seizures threshold changes up to 120 min after tDCS. In contrast, a stimulation of 200 μA resulted in significant effects even after only 30 min of stimulation.

Besides changes in seizure activity or epileptiform discharges, studies show either non-significant or very small changes in animal behavior during tES stimulation. Zobeiri and van Luijtelaar (2013) show a small increase in rats behavioral activity as recorded by passive infrared registration, but only for 150 μA and not 100 μA stimulation. Concerning the cognitive changes possibly induced by tDCS, Kamida et al. (2011) found that long-term tDCS treatment (30 min daily sessions for 2 weeks) rescued spatial memory impairment, measures *via* the Morris water maze test, in a rodent model of status epilepticus. Regarding the impact of tES on brain tissue, no histological abnormalities were found (Liebetanz et al., 2006; Zobeiri and van Luijtelaar, 2013). However, various molecular changes have been reported after tES. Importantly, several studies demonstrated a reduction in hippocampal mossy fiber sprouting (Kamida et al., 2011; Wu et al., 2020), associated with seizure severity and cognitive impairment after status epilepticus induction. Since seizures can lead to microglia activation and thus produce inflammatory cytokines, Regner et al. (2020) investigated the immunomodulatory effects of cathodal tDCS. Indeed, tDCS induced changes in interleukin 1 beta (IL-1β, one of the main pro-inflammatory cytokines) and Tumor Necrosis Factor alpha (TNF-α) levels. While IL-1β decreased in the hippocampus and increased in the cortex of cathodal tDCS treated animals, TNF-α displayed changes only at cortical level (increase in the cathodal tDCS group, decrease in the anodal tDCS group). Moreover, cathodal tDCS alone or associated with diazepam increased the cortical nerve growth factor (NGF) and brain-derived neurotrophic factor (BDNF) levels, which might be linked to the improved convulsive behavior of the tDCS-treated animals (Regner et al., 2020). Oppositely to cortical BDNF, hippocampal BDNF levels were reduced after cathodal tDCS treatment (Regner et al., 2020; Wu et al., 2020). Decrease of BDNF was moreover correlated to reduction of seizure severity (Wu et al., 2020).

Two studies moreover investigated the effects of tACS on epileptogenic activity *in vivo*. Berényi et al. (2012) showed the ability of standard 1 Hz tACS to modulate multi-unit activity firing and spike amplitude in a thalamocortical epilepsy rodent model. However, such open loop stimulation was unable to modulate spike and wave duration. Approaches using trigger-based stimulation, for instance sending a train of stimulation pulses when the system automatically detects a spike and wave pattern (closed loop stimulation), might be more efficient in modulating epileptogenic events. Closed loop tACS stimulation could indeed efficiently reduce spike and wave episodes duration by more than 60% with respect to sham (Berényi et al., 2012; Kozák and Berényi, 2017). Again, this effect is highly intensity-dependent: only a 10 V/m tACS, but not a 0.8 V/m one, could successfully modulate spike and wave patterns. Although applying an intracranial EF intensity of 10 V/m would pose safety concerns in humans, this study also shows that tES starting from 1 V/m can effectively entrain neurons by phase modulation. Nevertheless, it is important to notice that such closed loop studies used a triphasic stimulus pulse, which is hard to use in clinical settings for humans and needs high stimulation intensity to be effective. Although the closed loop treatment was highly effective in reducing time spent in seizures and seizure durations, its effects did not outlast the stimulation period, and the parameters that were effectively controlled by tES returned to baseline right after the end of the treatment (Kozák and Berényi, 2017).

Furthermore, since epilepsy is a chronic disease, understanding the long-term effects of tES is fundamental to better shape a stimulation paradigm for epileptic patients. Despite this, most of the studies investigated acute tES effects. Zobeiri and van Luijtelaar (2013) partially answered this issue by adding an EEG recording 24 h after 1 day of tES stimulation (4 sessions of 15 min cathodal tDCS) but found no difference in number or duration of spike and wave events compared to baseline pre-tDCS (Zobeiri and van Luijtelaar, 2013). Real long-term effects of tES were evaluated by Kozák and Berényi (2017) in an unsupervised closed loop tES experiment. When tES was applied chronically on demand for 6 weeks continuously (4 months and half for one rat), the decrease of seizure duration was constant over the weeks but returned to baseline right after the end of the treatment. The long-term effects of tES may as well depend on the time in which stimulation is applied. Indeed, Wu et al. (2020) found only acute effects when applying stimulation on epileptic rats but reported longer-lasting effects (up to few days after kainic acid injection) when tDCS was applied before the induction of epilepsy. Thus, tDCS might establish neuroprotective mechanisms which could have more long-term effects than stimulation protocols aimed at reducing ongoing seizure-like activity.

Overall, *in vivo* models of tES and epilepsy solve some of the issues of *in vitro* models, such as the need for more realistic brain anatomy and EF intensities. On the other hand, they still present some limitations. For instance, most of the studies here reported used epicranial electrodes (fixated onto the temporal bone) instead of transcranial electrodes (Liebetanz et al., 2006; Kamida et al., 2011; Berényi et al., 2012; Zobeiri and van Luijtelaar, 2013). This, coupled to relatively strong EF intensities and the differences in brain anatomy (rodents have smaller brains and lack gyrification), pose issues to the translation to human studies. Computational models of tES can provide partial solutions to such issues by using realistic human brain models and stimulation parameters.

#### Human studies: Electrical fields distributions

Although *in-vitro* and *in-vivo* studies have fully demonstrated the ability of weak electric fields to affect the firing patterns and excitability of neuronal populations, the study of tES mechanisms in patients with epilepsy is not straightforward. Indeed, to elucidate the underpinnings of weak EF in human brains, invasive recording techniques and innovative computational simulations are needed. A great step toward the understanding of EF propagation in the human brain has been made by tACS studies in DRE patients, using intracranial electrodes to measure the effects of stimulation in deep structures.

Thanks to such invasive recordings, tACS stimulation has been found to produce EF up to about 0.4 V/m when applying a 1 mA current through two scalp electrodes (Opitz et al., 2016; Huang et al., 2017; Louviot et al., 2022). Importantly, it has been shown that EF can reach structures as deep as the hippocampus and the amygdala (Huang et al., 2017; Lafon et al., 2017; Louviot et al., 2022). In such structures, Louviot et al. (2022) recorded maximal EF magnitudes of 0.38 and 0.49 V/m, respectively. Generally, EF peak intensities were achieved underneath the stimulation electrodes, but also in deep midline structures such as the anterior cingulate cortex (Huang et al., 2017; Lafon et al., 2017).

Reported EF magnitudes differ between records. Indeed, the measured EF in SEEG electrodes highly depends on the direction of the electrode with respect to the local EF vector and the way in which the EF is calculated. Although current models suggest that the EF magnitude should not depend strongly on frequency (for frequencies below a few kHz, Ruffini et al., 2013), Opitz et al. (2016) described an inverse correlation between frequency and EF magnitude, while the correlation was direct in Louviot et al. (2022). Indeed, the former study reported a 10% drop in EF magnitude at 150 Hz, the latter found that EF magnitudes at 1 Hz were 15% lower than at 300 Hz. Such discrepancies may be explained by instrumental or experimental conditions. Lafon et al. (2017) found that tACS at 2.5 mA peak intensity—above the 2 mA maximal current normally used on human subjects—gives rise to a maximal electrical field intensity of only 0.16 V/m across recording electrodes. Including details such as anisotropic white matter and inhomogeneous bone compartments does not improve prediction performance (Huang et al., 2017).

In conclusion, these studies not only demonstrate the ability of tES in reaching deep brain structures in human brains, but they also largely validate realistic modeling of electric fields. Since non-epilepsy *in vitro* models found that EF intensities of 0.2–0.5 V/m are sufficient for modifying spike timing and excitability (see *in vitro* chapter and Liu et al., 2018), the EF intensities found in the human brain would be able to modify neural activity even at 1 mA stimulation (Opitz et al., 2016; Huang et al., 2017; Louviot et al., 2022). Moreover, such reports confirm the importance of using computational models and provide important information for their future improvement. Nevertheless, there still are limitations. Firstly, the location of intracranial electrodes is sparse and corresponds to clinical needs, not to research ones. This restrains the measurements only to specific brain locations (mainly the ones involved in epilepsy) and prevents recordings from the brain surface, where the highest field intensity is expected. Secondly, there is no possible evaluation of differences between epileptic and healthy brains, restricting these results only to patients with epilepsy. The lack of cognitive, molecular, and pharmacological characterizations of such simultaneous tACS-intracranial recordings studies makes it hard to extrapolate the pure physical effects of field distributions from the physiological perturbations of epilepsy and cognitive states.

#### Computational models

In recent years, the development of mathematical and computational tools has allowed for the modeling of electric fields on realistic brain representations. Using biophysical models, various parameters of tES stimulation and brain composition may be analyzed, enabling to target brain regions with more accuracy (Sadleir et al., 2012). Computational head models allow for analysis of EF intensities and electrode positions which optimize the stimulation efficacy taking into account the unique geometry and conductive properties of individual heads. In this section, we review the main studies aimed at improving and clarifying the underpinnings of tES stimulation protocols in epilepsy patients *via in silico* models (Denoyer et al., 2020; Giannakakis et al., 2020). We summarize the findings of studies which analyzed the optimal stimulation montage parameters for reaching the highest EF spatial focality and intensity on target brain areas (Datta et al., 2009; Miranda et al., 2013; Parazzini et al., 2014; Ruffini et al., 2014; Opitz et al., 2018). Individualized head models are fundamental for defining the optimal stimulation montage. Even small deviations between the simulated and real brain target can in fact result in strong differences in the EF distributions applied during tES stimulation (Opitz et al., 2018). Moreover, modeling the presence of skull defects and the precise location of subdural or intracranial electrodes leads to significantly higher similarity between the predicted and the measured intracranial EF distribution (Opitz et al., 2018). In addition, the patient-specific geometry of sulci and gyri is crucial to evaluate the correct EF components on the brain surface (Miranda et al., 2013).

Using intracranial recordings during tES stimulation in 10 DRE patients, Huang et al. (2017) demonstrated that the possibility of reaching deep targets (as described in human subsection) might be possible thanks to the proximity of cerebrospinal fluid (CSF)-filled ventricles. Indeed, CSF is hypothesized to drive part of the skull-shunted current into deep brain regions (Datta et al., 2009; Huang et al., 2017). It is thus critical to integrate computational models of tES with CSF distribution and quantify its effect on EF distribution. Indeed, models accounting for this information found different EF distributions in the interfaces of gray matter with either white matter or CSF (Miranda et al., 2013). This result depended on the chosen conductivity values of white matter and CSF, insisting on the need of precise definition of conductivity values for better representing tissue heterogeneity. Studies on simultaneous tACS stimulation and invasive recordings in DRE patients or non-human primates also evaluated the accuracy of their individualized computational models of EF distributions. The simulations resulted in similar distributions than the actual recorded ones, with an accuracy of *r* = 0.86 for cortical electrodes and *r* = 0.88 for depth electrodes (Huang et al., 2017). It is nevertheless important to notice that, in some locations and on average, the simulated fields were higher than the real ones because of the limited spatial sampling of intracranial electrodes (median values across all electrode locations of 0.08 V/m in contrast with 0.002 V/m for real data) (Lafon et al., 2017; Opitz et al., 2018), or because of potentially wrong conductivity values (Huang et al., 2017; Opitz et al., 2018). The effects of tDCS depend on its polarity (Ghai et al., 2000; Lian et al., 2003; Bikson et al., 2004; Chang et al., 2015). Thus, it is crucial to model the EF orientation to best predict the effects of stimulation. Indeed, although EF magnitude indicates the strength of the induced field, the same magnitude applied to different brain regions (thus, neuronal composition and orientation) can give rise to different tES effects. For these reasons, Miranda et al. (2013) modeled the EF components during a bipolar DC stimulation (1–2 mA), finding that the highest values of the EF normal component concentrate in the sulci just below the stimulating electrodes, whereas the highest values of the tangential component occur on the gyri between the cathode and anode electrodes. Taking this into account, Ruffini et al. (2014) designed a computational model of possible tES montages aimed at maximizing the chosen EF component on the target areas. Such an approach appears pivotal for the optimization of tES clinical effects and the characterization of the differential roles of the EF components.

Another important parameter in the efficacy of tES stimulation is the geometry and position of scalp electrodes. Although the most used electrodes were, until recently, large (25 cm^2^ or more) bipolar patches, this solution might not optimize the spatial control and focality required for epilepsy treatment. A configuration using small (0.4 cm radius) electrodes in a ring is more suitable for reaching the desired focality of stimulation beneath the stimulating electrodes, avoiding potentially unwanted stimulation of non-targeted areas (Datta et al., 2009). Nevertheless, despite the greater focality given using smaller electrodes, this configuration needed two times the current used with a rectangular patch to produce the same EF intensity in the targeted area. This highlights the importance of studying other electrode configurations and dimensions to achieve focality without the need of increasing the current intensity. To answer these questions, Ruffini et al. (2014) studied 26 different configurations of multifocal montages with small electrodes (1 cm radius, anode at Cz and cathode at one of the other 26 positions of the 10–20 system) and compared the results with a model using two circular sponge electrodes (25 cm^2^). With the goal of defining the optimal montage to target extended brain areas, they used precise head models combined with detailed cortical target maps obtained by functional data such as resting-state fMRI, PET, and EEG. Due to the complex nature of drug-resistant epilepsy, assigning target weights to specific cortical regions can strongly improve the tES effects, for instance by differentially optimizing the magnitude of the normal or tangential components of the EF according to the individual cortical anatomy (Ruffini et al., 2014). This study also confirmed the improvement of stimulation spatial focality when using multifocal montages with small electrodes vs. large-sponge bipolar montages (Figure 1A).

Additionally, the anode position in tDCS clinical protocols has been a matter of discussion (Bikson et al., 2016). Although most montages with two electrodes place the anode on non-epileptogenic brain areas, some protocols (Auvichayapat et al., 2013; Lin et al., 2018) place it over the contralateral shoulder. Parazzini et al. (2014) used customized brain models to demonstrate that varying the anode position induces different EF intensity distributions not only at cortical level but also in the deeper brain regions. More specifically, they showed that placing the anode over the shoulder significantly enhances the EF induced in deep brain regions (including thalamus and hippocampus) and strongly increases the spatial focality of cathodal stimulation with respect to an anode positioned on the contralateral cortex. Therefore, the risk of seizure induction in epilepsy patients during tDCS might be limited using an extracephalic anode, which could dramatically reduce the excitability levels induced beneath the anodal electrode. However, placing the anode on the upper arm could cause hardly predictable changes in the current flow, increasing the amount of current flowing in parietal regions even when the parietal cortex is not directly targeted. Moreover, extracephalic electrodes might lead to spurious currents in vital areas such as the heart, brainstem, and respiratory system (Vandermeeren et al., 2010). More safety and electric fields distribution studies are needed before employing extracephalic electrodes in a more consistent manner. Correct electrode placing is crucial; indeed, electrodes should be placed a minimum of 1 cm away from the optimal location given by simulations to achieve the predicted EF distribution (Opitz et al., 2018). For more information on the possible implementation guidelines of tDCS, we refer the reader to the review by Thair et al. (2017).

In addition to providing useful information on EF distribution and optimal stimulation design, computational models allow to characterize the physiological mechanisms of tES on brain activity. To model the long-term effects of tDCS on epileptic activity, Giannakakis et al. (2020) investigated how the brain structural connectivity prior to stimulation can affect the changes induced during and after a stimulation session. With this aim, they used structural connectivity data from healthy and epileptic patients to create either healthy or epileptic network nodes, modeling tDCS effects up to 24 h after stimulation. The main findings were: (1) the simulated effects of tDCS differ between network models of either healthy controls and epilepsy patients; (2) the modeled stimulation of epileptogenic networks resulted in medium-term (5–6 h), connectivity decrease inside the targeted areas and in long-term connectivity increase inside some non-stimulated regions connected to the stimulated ones; (3) there was a high inter-subject variability in both short- and long- term effects of tDCS, given by the individual pre-stimulation connectivity matrices. Interestingly, when comparing the simulated data with the clinical outcomes of the patients used to create such models, there was a weak correlation between the connectivity increase in non-targeted areas and worsen surgery outcome. It is important to notice that the tDCS session was modeled as a 50% reduction of external input in the simulated amygdala, hippocampus and parahippocampus nodes. Despite the ability of tES in reaching such deep brain regions, the EF intensities found there correspond to a maximum of 0.49 V/m (see the paragraph on EF distributions and Huang et al., 2017; Lafon et al., 2017; Louviot et al., 2022). It is thus arguable that tES could result in a decrease of deep structures activity of 50% with such a small EF modulation, but more studies are needed to define the precise activity reduction corresponding to the real EF intensities measured. Another computational study tried to shed light on the potential cellular mechanisms responsible for both acute and long-lasting effects of cathodal tDCS on epileptic activity (Denoyer et al., 2020). To this aim, they developed a novel approach of computational models combining both cellular and neural mass features, designing an *in silico* thalamocortical column based on physiological data. To study the plasticity effects of tDCS, they inserted in the model glutamatergic excitatory cells and three types of GABA-ergic interneurons. In this model, the immediate effects of cathodal tDCS on a large (10.000 neurons) cortical column resulted in a small firing rate reduction in all cell types and tDCS effects were best explained by modulation of the presynaptic probability of release. The firing modulation was stronger in vasointestinal peptide expressing (VIP) interneurons, which are mostly present in the upper cortical layer. Regarding long-lasting effects, their model suggested that the decrease in epileptiform activity given by simulated cathodal tDCS is highly influenced by the network size. More specifically, large networks of 10 thousand neurons showed the longer-lasting effects on ED decrease, while small networks of 250 neurons only showed immediate effects. Recently, following the work of Wendling et al. (2002) and Lopez-Sola et al. (2021) provide a laminar NMM capable of realistically reproducing the electrical activity recorded by SEEG in the epileptogenic zone during interictal to ictal states. A novel element in the model is a physiologically motivated algorithm for chloride dynamics: the gain of GABAergic post-synaptic potentials is modulated by the pathological accumulation of chloride in pyramidal cells due to high inhibitory input and/or dysfunctional chloride transport. By integrating pathophysiological mechanisms, such models can provide a better basis to understand the effects of tES EFs and optimize stimulation protocols.

In conclusion, computational models have been increasingly used for the design of tES protocols and showed crucial results for the optimization of stimulation montages targeting focal or extended brain regions. Within recent years, the creation of complex, personalized head and brain models resulted in the precise investigation of EF distributions inside the brain and the comparison with real data from which these models were generated. Moreover, the integration of personalized models with high resolution anatomical imaging (e.g., MRI, CT scan, and DTI) and with physiological data (e.g., EEG, iEEG, and PET) has allowed the design of high-resolution models (up to 1 mm^3^) informed by patient-specific functional information on the epileptogenic network.

Nevertheless, there are still some main limitations. First, even though the studies highlight the importance of CSF in the EF propagation across deep brain areas, there is little account for CSF simulation parameters and distribution in individual head models. Additionally, the used conductivity values were almost exclusively based on relatively old *ex-vivo* studies, underlying the need for a better characterization of conductivity values based on *in vivo* studies. The further step in this direction would be the use of individual conductivity measures, especially important for epilepsy (Huang et al., 2017; Opitz et al., 2018). Although it is generally thought that the neurons are influenced only by the EF component parallel to their axo-somatic axis (Bikson et al., 2004), the precise impact of tangential and normal EF components on small neuronal networks in different brain regions has yet to be unraveled (Miranda et al., 2013; Ruffini et al., 2014). This is even more problematic when considering that cortical pyramidal neurons are mostly perpendicular to the cortical surface, but their axonal projections and cortical interneurons are often aligned tangentially to the surface (Fox et al., 2004). Characterizing such effects is of fundamental importance for designing optimal stimulation montages depending on the local brain anatomy and axon orientation.

## Discussion

We performed a systematic review of clinical and fundamental studies using tES as a therapeutic approach in epilepsy. First, clinical cases and clinical trials performing cathodal tDCS in patients suffering from DRE provided preliminary encouraging results regarding the clinical improvement of these untreatable patients, estimated by seizure frequency (SF) changes. Most studies (14/16 of clinical trials) reported a decrease in SF after one or several sessions of tDCS in patients suffering from DRE, with cumulative effects, compared to the baseline SF. Thus, one of the main clinical challenges of this technique is to prolong the beneficial after-effect due to tDCS. Studies with long-term follow-up interestingly showed that the repetition of stimulation and the length of the duration of the break between two stimulations are of major importance (Monte-Silva et al., 2010; Yook et al., 2011; Yang et al., 2020; Kaufmann et al., 2021). When tDCS is applied for several consecutive days, the resulting decrease in SF lasted for several weeks or months (Auvichayapat et al., 2016; Tekturk et al., 2016a,b; Lin et al., 2018; Yang et al., 2019, 2020; Kaye et al., 2021). Another issue of using neuromodulation techniques as epilepsy treatment is to specifically target the EZ. In this view, the development of multichannel tDCS or HD-tDCS using several pairs of smaller electrodes than the two large electrodes conventionally used showed promising results. Multichannel tDCS makes it possible to optimize and personalize the electrode montage to target more precisely the epileptic focus while sparing the rest of the cortex from any effect (Ruffini et al., 2014; Kaye et al., 2021; Daoud et al., 2022). Moreover, the use of more channels enables to target multiple foci and complex and complex brain networks distributed over wide regions of the cortex (Sanchez-Todo et al., 2018). Another interest of using multichannel tDCS is the potential ability to reach deeper brain regions. Indeed, one of the main limitations of conventional tDCS is the very superficial diffusion of electrical field with the use of two large sponge electrodes and a very weak electrical current, making very difficult to reach deep brain structures. Recent evidence has shown that optimizing stimulation montage with several electrodes allows to achieve more focal stimulation for superficial targets and reach deep targets with more intense stimulation, mostly due to cerebro-spinal fluid guiding currents deep into the brain (Huang and Parra, 2019). An indirect modulation of deep brain regions might also be achieved targeting their related cortical functional brain network, as proposed by Fox et al. (2014). They used common targets of deep brain stimulation as seeds in functional connectivity-resting state MRI, to obtain a functional representation of the cortical areas positively or negatively correlated to them. Multichannel tES can be optimized to enhance positive or negative regions, inducing changes in their connectivity with the seed and possibly modulating its activity.

Moreover, regarding the safety of tES, no serious adverse effects have been reported after application of tDCS in both children and adults with epilepsy. The most common effects were the slight tingling and itching sensation during the stimulation. The use of tACS in epilepsy is promising but relatively under-examined. The safety of this technique has been assessed in epileptic patients (Opitz et al., 2016; Lafon et al., 2017) and a recent study obtained interesting preliminary studies of tACS in epilepsy (Holmes et al., 2019). To design optimal stimulation protocols for patients, it is necessary to investigate the underlying physiological mechanisms of tACS, *via* cell cultures, animal, or computational models. *In vitro* studies have demonstrated that the acute and long-term effects of tACS can differ from the ones observed in tDCS (Lian et al., 2003; Bikson et al., 2004). Nevertheless, this technique is often neglected in most *in vivo, in silico*, and clinical studies. Since the working hypothesis of tACS is that it can disrupt or amplify endogenous brain rhythms (Berényi et al., 2012; Lafon et al., 2017), it will be required to design individual protocols based on brain signals recordings for the definition of precise target frequencies. The use of online EEG, iEEG or MEG data, coupled with *in silico* studies using personalized head models and cortical target maps, might allow the closed loop tACS stimulation of epilepsy patients (Ruffini et al., 2014).

Furthermore, electrophysiological measures are used to quantify the clinical outcome resulting from tDCS application. The most common technique applied in clinical trials studying tDCS effects on epileptic patients is to measure changes in interictal epileptiform activities using electrophysiological data (EEG and/or MEG). However, this approach is questionable because interictal activity is not a reliable marker of epilepsy efficacy or severity. The discrepancies observed between the different studies in this field led to open the path to new brain activity measures more suited for studying plasticity changes induced by tDCS. Indeed, considering epilepsy as a brain network disease, analyzing the functional alterations due to tDCS is highly relevant as a marker of epilepsy improvement. Recent studies focusing on functional brain network changes have highlighted interesting results between the response to tDCS therapy and functional disturbances in global brain networks (Lin et al., 2018; Tecchio et al., 2018; Daoud et al., 2022). Measuring FC changes may thus be a possible predictor of the tES clinical efficacy in epileptic patients (Hao et al., 2021). However, measuring changes in functional connectivity after tDCS in epilepsy has some limitations. One of the issues in studying FC changes is that DRE patients are taking several and different pharmacological treatments. Although they remained unchanged during the studies, they could affect the FC and the efficacy of tDCS. In addition, most studies using electrophysiological data measuring FC have employed EEG with low numbers of EEG channels, limiting the accuracy of localizing brain sources affected by functional changes. Finally, the functional changes observed over long periods of time are not known and this requires future studies on the long-term effects of tDCS. Thus, network effects are no doubt important, but they necessitate computational models combining physics and physiology to evaluate mechanistic implications and properly interpret data. In this view, by unraveling the underlying cellular and molecular mechanisms involved in the tES-induced electrophysiological plasticity, further studies could lead to more realistic computational models and to improved clinical stimulation protocols.

The reported physiological studies have demonstrated that tES induces concurrent and plastic effects that are relevant to epilepsy and its control. Overall, *in vitro* studies suggest that acute effects of both tDCS and tACS are likely related to membrane polarization (Bikson et al., 2004), with a major influence on spike timing and spike-timing-dependent plasticity (Reato et al., 2010; Rahman et al., 2017). However, stimulation effects are not limited to polarization. Crucial factors underlying tDCS are as well NMDA receptor-mediated synaptic strength modulation (Liebetanz et al., 2006; Chang et al., 2015; Sun et al., 2020); reduction of mossy fiber sprouting (Kamida et al., 2011; Wu et al., 2020) and of hippocampal cell loss (Kamida et al., 2011); modulation of GABAergic cortical inhibition (Dhamne et al., 2015); modulation of neuroinflammation, neurotrophines, IL-1β, TNF-α, NGF, and BDNF (Regner et al., 2020). Regarding tACS effects, studies have moreover showed a stimulation-related increase of extracellular K+ (Bikson et al., 2001; Lian et al., 2003), which might reflect a depolarization block. Indeed, the persistent membrane depolarization would lead to tonic inactivation of Na+ channels, which in turn would lead to action potential's initiation. Another hypothesis on the mechanisms of tACS is that it could recruit subset of thalamic cells, which would be in their refractory phase during the spike and wave duty cycle, thus preventing the synchronous cell firing of the ongoing rhythm (Berényi et al., 2012; Kozák and Berényi, 2017).

Later and sustained effects may be related to neurogenesis, cortical reorganization and likely to changes in synaptic plasticity (Chang et al., 2015; Rahman et al., 2017; Sun et al., 2020). To better characterize tDCS effects on epilepsy, future studies should differentiate the pathophysiological plasticity induced by the epileptic activity itself leading to hyperexcitability (e.g., pathological LTP and NMDA receptor upregulation), and the physiological mechanisms of plasticity that might be induced by tDCS. It is unclear whether *in vitro* and *in vivo* findings obtained with EF magnitudes higher than 1 V/m are representative of real tES protocols. Most *in vitro* DC stimulation studies have so far involved electric field magnitudes of at least 20 V/m (20 times higher than the EF induced in the human brain for a 2 mA tDCS protocol). Even with the EF magnitudes used by Ghai et al. (2000) and Chang et al. (2015) (2–16 V/m), the membrane voltage changes induced by either DC or AC fields at low amplitudes are consistently lower than the action potential firing threshold. Therefore, one needs to investigate how this effect can be amplified to modulate large networks. One hypothesis is that tDCS can increase the presynaptic firing rate and modulate spike timing, increasing synaptic integration and thus the coincidence of pre- and post-synaptic APs (Rahman et al., 2017). However, it is not clear how might result in brain-wide effects as it happens during *in vivo* stimulation. To understand this, it is crucial to model the effects in neuronal network models. Although not included in most of the studies reported here, the endogenous neural activity is essential for obtaining realistic tES effects, as proved by the studies of Ghai et al. (2000) and Chang et al. (2015). The interaction between of tES exogenous fields with endogenous field mechanisms can give rise to further amplification effects, which could cause important firing activity changes at the network level (Fröhlich and McCormick, 2010; Weiss and Faber, 2010). Future *in vitro, in vivo*, and computational studies should thus model endogenous field effects (e.g., ephaptic coupling) in order to better characterize the combined endogenous field and tES network effects (Fröhlich and McCormick, 2010; Reato et al., 2010; Ruffini et al., 2020).

Furthermore, the present studies emphasize the need for patient-tailored models, including functional and structural connectivity data (Ruffini et al., 2013, 2018; Tecchio et al., 2018), precise anatomical reconstruction with skull defects and CSF models (Datta et al., 2009; Huang et al., 2017; Louviot et al., 2022), metal implants (Mercadal et al., 2022) as well as detailed target mapping (Ruffini et al., 2014). The strong inter-subject variability of tDCS-induced connectivity changes could cause the variability in the effectiveness of stimulation (Giannakakis et al., 2020). The recent technique of transcranial Individual Neurodynamics Stimulation (tIDS), which mimics the endogenous dynamics of the target neuronal networks, might partially answer this issue through the characterization of individual local functional connectivity prior to stimulation (Cottone et al., 2018). Other techniques also aim at restricting the tES spatial focus (for a recent review, the reader is referred to Takeuchi and Berényi, 2020). For instance, Intersectional Short Pulse Stimulation (Vöröslakos et al., 2018) and concentric ring electrodes montages (Besio et al., 2007) increase spatial focality of tES and may be useful when the target area is confined to a small area. However, they do not improve tES ability to target deeper structures. The novel technique of Temporal Interference (TI) could tackle this issue by creating a constructive interference of high-frequency transcranial stimulation waves. Consequently, TI might reach deeper structures such as the hippocampus (Grossman et al., 2017; Missey et al., 2021), but it is still unclear how amplitude modulated kHz electric fields influence the areas inside or outside the target.

## Data availability statement

The original contributions presented in the study are included in the article/supplementary material, further inquiries can be directed to the corresponding author.

## Author contributions

FB, MD, and SS conceived the review idea. MD and SS performed independently the literature search, compared their final report selection, and wrote the manuscript. FB reviewed the manuscript and the selection process. MCB designed and created Figure 1. C-GB, MCB, FW, PB, and GR contributed to the manuscript critical revision. All authors contributed to the article and approved the submitted version.

## Funding

This work was supported by the European Research Council: Galvani Project, ERC-SyG 2019, Grant Agreement No. 855109.

## Conflict of interest

Authors GR and MCB were employed by Neuroelectrics Barcelona. The remaining authors declare that the research was conducted in the absence of any commercial or financial relationships that could be construed as a potential conflict of interest.

## Publisher's note

All claims expressed in this article are solely those of the authors and do not necessarily represent those of their affiliated organizations, or those of the publisher, the editors and the reviewers. Any product that may be evaluated in this article, or claim that may be made by its manufacturer, is not guaranteed or endorsed by the publisher.
